# Explainable Transformer-Based Framework for Glaucoma Detection from Fundus Images Using Multi-Backbone Segmentation and vCDR-Based Classification

**DOI:** 10.3390/diagnostics15182301

**Published:** 2025-09-10

**Authors:** Hind Alasmari, Ghada Amoudi, Hanan Alghamdi

**Affiliations:** Department of Information Systems, Faculty of Computing and Information Technology, King Abdulaziz University, Jeddah 21589, Saudi Arabiagaamoudi@kau.edu.sa (G.A.)

**Keywords:** glaucoma, vision transformers (ViTs), transfer learning, vertical cup-to-disc ratio (vCDR), fundus images, U-Net

## Abstract

Glaucoma is an eye disease caused by increased intraocular pressure (IOP) that affects the optic nerve head (ONH), leading to vision problems and irreversible blindness. **Background/Objectives**: Glaucoma is the second leading cause of blindness worldwide, and the number of people affected is increasing each year, with the number expected to reach 111.8 million by 2040. This escalating trend is alarming due to the lack of ophthalmology specialists relative to the population. This study proposes an explainable end-to-end pipeline for automated glaucoma diagnosis from fundus images. It also evaluates the performance of Vision Transformers (ViTs) relative to traditional CNN-based models. **Methods**: The proposed system uses three datasets: REFUGE, ORIGA, and G1020. It begins with YOLOv11 for object detection of the optic disc. Then, the optic disc (OD) and optic cup (OC) are segmented using U-Net with ResNet50, VGG16, and MobileNetV2 backbones, as well as MaskFormer with a Swin-Base backbone. Glaucoma is classified based on the vertical cup-to-disc ratio (vCDR). **Results**: MaskFormer outperforms all models in segmentation in all aspects, including IoU OD, IoU OC, DSC OD, and DSC OC, with scores of 88.29%, 91.09%, 93.83%, and 93.71%. For classification, it achieved accuracy and F1-scores of 84.03% and 84.56%. **Conclusions**: By relying on the interpretable features of the vCDR, the proposed framework enhances transparency and aligns well with the principles of explainable AI, thus offering a trustworthy solution for glaucoma screening. Our findings show that Vision Transformers offer a promising approach for achieving high segmentation performance with explainable, biomarker-driven diagnosis.

## 1. Introduction

Glaucoma is an ocular disease that causes irreversible blindness due to damage to the ONH resulting from increased pressure in the eye. The World Health Organization (WHO) has reported that glaucoma is the second leading cause of vision loss in the world after cataracts [1]. It represents a major global health concern, as the worldwide estimate indicates that the number of adults with glaucoma in 2013 was 64.3 million [2], increasing to about 78 million in 2020, and it is expected to reach 111.8 million by 2040 [3].

Due to the large increase in the number of people expected to develop glaucoma, the demand for ophthalmologists has increased, as there is a shortage of workforce in ophthalmology, which is one of the problems addressed to solve worldwide. Since glaucoma does not show any symptoms in people who have it [2,3], screening-based treatment for early detection of glaucoma is crucial. Therefore, this process relies heavily on the availability of specialists to carry out complex procedures by analyzing image results, which is time-consuming and could lead to delays in diagnosis that affect the early detection and prevention of vision loss [4,5]. To detect the disease, ophthalmology specialists can perform several tests, including Visual Fields (VF), Optical Coherence Tomography (OCT), and fundus images [2]. In this research, we focus on fundus images due to their availability in most hospitals and because they are less expensive than other devices [6]. Fundus images provide information about the optic disc (OD), optic cup (OC), blood vessels, macula region, and fovea that are used in diagnostic procedures.

- Optic Nerve: Responsible for transmitting visual information from the retina to the brain. It consists of millions of nerve fibers that gather in the optic disc, which is the beginning of the optic nerve [7].
- Optic Disc (OD): The point where nerve fibers come together to exit the eye, forming a circular area on the retina without photoreceptors, which makes it a blind spot in the visual field [7,8].
- Optic Cup (OC): The bright central part of the optic disc, surrounded by the neuroretinal rim, which consists of the remaining tissue of the optic nerve head. It is also considered a blind spot like an optical disc because it does not contain photoreceptors. Its size varies due to several factors, including increased intraocular pressure, one of the main parameters for detecting glaucoma [9].
- Blood Vessels: They supply the optic nerve head and retina with blood that contains oxygen and nutrients. When any abnormalities or changes occur in the blood vessels, they may be an indicator of eye diseases such as glaucoma [10].
- Macula Region and Fovea: The macula region is a round area located near the middle of the retina and has the highest percentage of photoreceptor cells and is responsible for central vision and visual acuity. In contrast, the fovea is the central portion of the macula, which contains a higher density of photoreceptor cells. It is responsible for sharp central vision and is essential for tasks requiring detailed visual discrimination [7,11].

**Figure 1:**
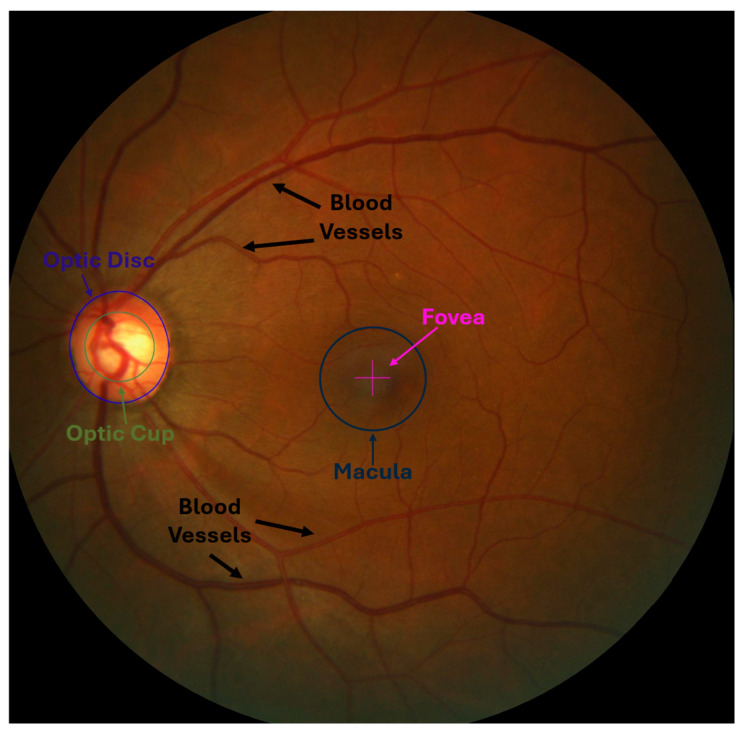
The fundus image shows fundamental anatomical structures: the macula region with the fovea at its center, blood vessels, and the optic disc and cup on the left.

Figure 1 illustrates the anatomical structures mentioned and their respective locations.

**Figure 2:**
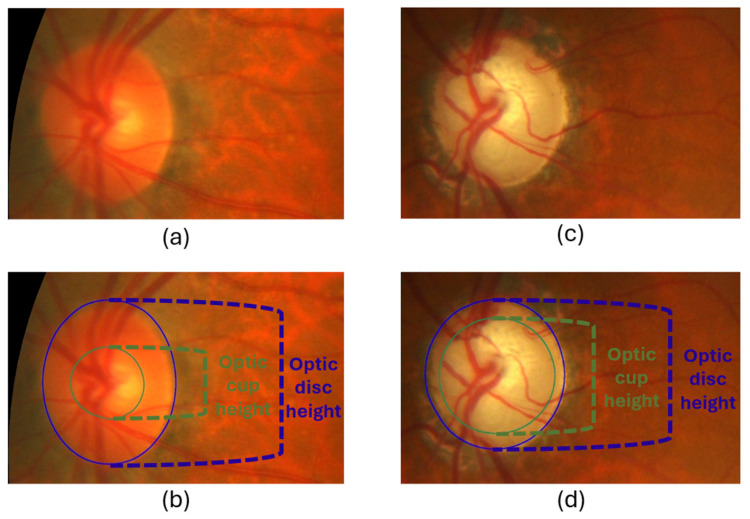
Cup-to-Disc Ratio for normal (**a**,**b**) and glaucoma (**c**,**d**).

One of the ways to detect glaucoma from a fundus image is by calculating the Cup-to-Disc Ratio (CDR), which is the ratio of the OC diameter to the OD diameter. A person with glaucoma has an enlarged OC diameter relative to the OD [12]. If the CDR value is less than 0.5, it is considered normal [13]; if the value is greater than 0.5, it is most likely to be considered glaucoma [13,14]. To see an illustrative example, see Figure 2.

The development of technology has brought about a significant transformation in many fields, including medicine. The use of machine learning (ML) and deep learning (DL) in recent years has made a significant difference in the early detection of glaucoma [15]. Machine learning is a branch of artificial intelligence (AI) that focuses on designing and developing algorithms to create a machine capable of learning and making decisions based on data, patterns, and previous experiences without the need to program it. However, intervention is still needed to convert raw data into features that can be used as input. Because a machine is not capable of learning features on its own, there is a need for domain knowledge. For example, when using ML to detect glaucoma, specialists specify parameters that are used as input features. However, there is no guarantee that the parameters chosen will give the best results [16].

This is where deep learning, a subfield of machine learning, plays a role. It focuses on developing an artificial neural network that mimics the work of the human brain. It can work on more complex matters without domain knowledge, as it can extract and learn from features. For example, fundus images can be provided as input without specifying features, and the model will extract them and use them to make predictions, which helps it discover unclear patterns and provide better results. Deep learning can extract good features if there are enough data to train on [17]. This may be a problem, as medical images are not available in sufficient quantities to train models from scratch. To solve the problem, transfer learning (TL) is used, a category of machine learning techniques that trains models on specific tasks or data to be reused on other data or tasks. This technique reinforces the results with previously acquired knowledge to solve the current problem. It has contributed significantly to several fields, including the medical sciences, specifically in medical imaging. Glaucoma detection is conducted through fundus images. Using one of the pre-trained convolutional neural networks (CNN) models that were trained on the ImageNet dataset, which contains more than a million diverse images in several fields, will achieve better results in a shorter time than starting from scratch. Many studies have been conducted to detect glaucoma using pre-trained models [18].

With recent developments in deep learning architectures, a transformer was built. It achieved significant success in natural language processing (NLP), inspiring developers to leverage it and use it in computer vision, which was previously dominated by convolutional neural networks (CNNs). Vision Transformers (ViTs) were proposed in 2020 by Dosovitskiy et al. [19]. They segment images into sequences of patches so that they can be treated as tokens in text. Transformers feature the ability to find long-distance relationships, which has been helpful in NLP for understanding text and linking relationships. It can also be used in images to find relationships across entire images rather than local relationships within an image, providing greater understanding and better results. Vision Transformers (ViTs) have been used in classification tasks and have achieved good results, opening the way for their use in object detection and segmentation tasks [19].

In 2021, the Swin Transformer, a new model designed to serve as a backbone for general use in computer vision tasks, was introduced. It can serve as a backbone for classification, object detection, and segmentation, like the role played by CNNs. The Swin Transformer also solves the problem that ViTs faced when dealing with high-resolution images and computational complexity that grows quadratically with image size. This high computational complexity arises because, as mentioned before, the entire image is divided into a sequence of patches. For each patch, self-attention is used to find the relationship between the patch and the rest of the patches in the entire image. To solve this, the Swin Transformer uses Window-based Multi-Head Self-Attention (W-MSA) and Shifted Window-based Multi-Head Self-Attention (SW-MSA) in the Swin Transformer block. Using W-MSA, images are divided into windows, each containing a set of patches. This method finds the relationship between each patch and the other patches present only in the same window, not the entire image. This reduces computational complexity and, therefore, results in linear computational complexity with image size. However, this limits contextual understanding. To solve this problem, SW-MSA is used, which moves or shifts the window to include different patches of the image and increases the understanding of the relationships between patches in other windows. Additionally, Swin starts with small patch sizes and then merges them with other patches, gradually increasing their size in the Patch Merging phase after each Swin Transformer block. Therefore, the Swin Transformer has a hierarchical nature [20].

The MaskFormer model was proposed as a unified model for use across different segmentation types. It starts by handling how each type of segmentation processes the mask: semantic segmentation uses per-pixel classification, while instance segmentation uses mask classification. Therefore, all masks of the same type are created by converting the per-pixel classification into a mask classification and processing it. This unified approach allows for handling semantic and instance segmentation tasks within a single framework.

MaskFormer consists of three modules. The first is the pixel-level module, which contains the backbone, such as ResNet, Swin Transformer, or other models, and the pixel decoder. The backbone’s job is to extract important features from the input image. After extracting features, the pixel decoder turns them into per-pixel embeddings that have a description of each pixel in the image. At the same time, we have a transformer module, which contains a transformer decoder that uses the features extracted from the image to produce N segment embeddings, each of which represents a description of a potential object or a potential region of one of the objects the model aims to segment. Finally, the segmentation module receives two inputs, pixel and mask embeddings, which it combines to produce binary masks for each object and a class prediction or class label for it [21].

Another computer vision task is object detection, which is also an important step in most tasks, as it helps improve the performance of other models. When choosing an appropriate model for object detection, some significant metrics to consider are the model’s accuracy in identifying the object based on its classification and its accuracy in determining the object’s localization, while also considering speed. The two have an inverse relationship: the higher the accuracy, the lower the speed, and vice versa. There are two types of techniques used in object detection: traditional techniques that have been used since the beginning of object detection, which rely on handcrafted features, and techniques that have evolved with the development of CNNs, which rely on deep learning. The second type, deep learning-based detection, has two types: two-stage and one-stage detectors [22].

Starting with two-stage detectors, as the name suggests, the detection process takes two steps, making the model take longer but achieving better overall accuracy. An example of this type is the RCNN model, which was proposed in 2014 and achieved good results compared to traditional object detection models. However, its drawback is slow detection speed, as it relies on selective search to generate a large number of region proposals and then extracts features from each proposal, which makes it time-consuming and computationally expensive. Then, Fast RCNN was proposed in 2015, which, as the name suggests, was an improved version of RCNN in terms of speed. With this model, they reduced the time spent generating predictions for each proposal and increased accuracy. However, it was still necessary to reduce the time spent creating proposals. To solve this problem, Faster RCNN was proposed in 2015, not long after Fast RCNN. It used a region proposal network (RPN) instead of selective search to reduce the proposal generation time and solve the previous problem. Faster RCNN is the first deep learning-based detection model to achieve near-real-time speed. However, the model is still computationally expensive and time-consuming compared to one-stage detector models, so it is not used in some fields [22].

On the other hand, one-stage detectors detect an object in one step, making them faster and more useful when real-time models are needed. They are often used in mobile devices, but their drawbacks include poor performance when detecting dense and small objects. The first one-stage detector model proposed was You Only Look Once (YOLO) in 2015. This model obtains the image proposal region and predicts bounding boxes with probabilities for each region in one step. It is known for its high speed, but compared to two-stage detectors, it has lower localization accuracy, especially for small objects. This has been improved in newer versions of the model, as there are several versions [22], the latest of which, YOLOv11, was released in 2024.

By leveraging technological developments in this field, this research aims to assist ophthalmologists in the early detection of glaucoma, reducing the effects of the disease and providing a better quality of life for patients. This research proposes an explainable end-to-end pipeline for automated glaucoma diagnosis.

## 2. Related Work

Early detection of glaucoma remains a critical challenge due to its asymptomatic progression and irreversible vision loss. Therefore, many previous studies developed Computer-Aided Diagnosis (CAD) systems that used machine learning and deep learning techniques to automate the detection process and enhance diagnostic accuracy. These systems typically determine the presence of glaucoma either by analyzing the vertical Cup-to-Disc Ratio (vCDR) resulting from segmentation or by directly processing the entire fundus image.

A traditional machine-learning approach was used by Jun Cheng et al. [23], based on superpixel classification and support vector machine (SVM), for segmenting the optic disc and cup. A rule-based glaucoma diagnosis was then applied by calculating the CDR. However, this method lacks generalization across different datasets and is sensitive to image artifacts.

With the development of deep learning techniques and their expanded use in computer vision tasks and medical fields, numerous studies have been conducted. Mamta Juneja et al. [24] used CNNs based on a modified U-Net architecture to sequentially segment the optic disc and optic cup from fundus images. Another approach by Huazhu Fu et al. [25] used a U-shaped CNN with multi-label learning to simultaneously segment the optic disc and optic cup and then classify them using vCDR.

Some studies have relied on traditional clinical biomarkers, such as the vCDR, for diagnosis. Alexandre Neto et al. [26] used the U-Net architecture with the Inception v3 model for segmenting the OD and OC and calculated the vCDR for classification. Other studies extract complex patterns and discriminative features directly from the image. Javier Civit-Masot et al. [27] used a generalized U-Net for segmentation and pre-trained transfer learning MobileNetV2 for classification. A voting scheme was used to obtain an ensemble network. Additionally, Vismay Agrawal et al. [28] used an ensemble approach in segmentation using two CNN models and Contrast Limited Adaptive Histogram Equalization (CLAHE)-enhanced inputs. The first model receives the RGB fundus images, and the other adds coordinate space (X and Y) to it. This approach leverages the segmentation output to crop image patches, which are then used as inputs for glaucoma classification, which uses DenseNet201 and ResNet18. Syna Sreng et al. [29] used the DeepLab v3+ model with MobileNet for segmenting OD and then used deep features extracted through transfer learning, SVMs, and ensemble methods for classification. However, this approach lacks transparency regarding the basis for the diagnosis due to the lack of an explanation.

To provide transparency, some studies have used Explainable AI (XAI) techniques. Vijaya Kumar Velpula and Lakhan Dev Sharma [30] used five pre-trained deep CNN models to detect glaucoma, followed by Classifier Fusion using the maximum voting-based approach (MVB). To clarify the results, they used visualization techniques such as gradient-weighted class activation mapping (Grad-CAM) and locally interpretable model-agnostic explanations (LIME), among others, to clarify the basis on which the models made their diagnoses.

Additionally, with the emergence of transformer technology and its subsequent development in the field of Vision Transformers, it has been applied to various tasks, beginning with classification and extending to multiple fields, including medicine. Farheen Chincholi and Harald Koestler [31] used the Vision Transformer (ViT) and Detection Transformer (DETR) in the tasks of object detection and glaucoma detection using the distance radius for bounding boxes of the OC and OD. Table 1 contains a summary of the related work.

In conclusion, based on a review of related work, there is diversity in the use of classification, segmentation, and object detection techniques. However, there is still a need to develop a pipeline for automated glaucoma that provides clarity and interpretation of the results obtained by the models. Our work aims to bridge this gap by proposing an explainable end-to-end pipeline for automated glaucoma diagnosis that includes object detection, automated segmentation of the OD and OC, and classification based on vCDR. Using vCDR will provide transparency and interpretation based on traditional clinical biomarkers. Moreover, we evaluate the effectiveness of ViTs in segmenting the OD and OC regions and assess whether they can outperform traditional CNN-based architectures such as U-Net.

## 3. Materials and Methods

**Figure 3:**
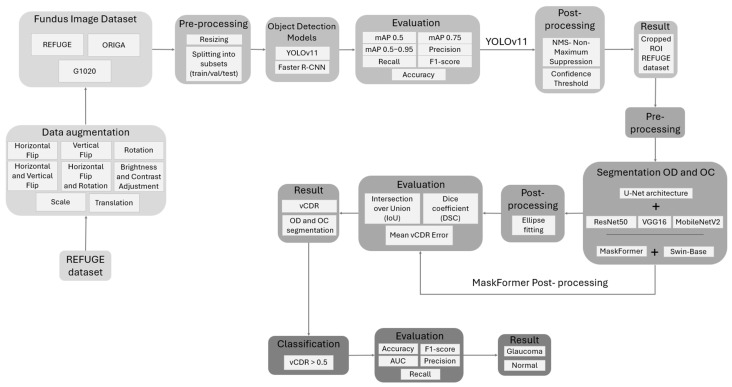
Overview of the proposed methodology.

This section contains four subsections: data collection, which includes the datasets used; the preprocessing utilized to prepare the datasets; the object detection stage; and the models used to generate the new dataset used in the segmentation and classification stages. Figure 3 illustrates the methodology employed in this study.

### 3.1. Data Collection

In this study, three public datasets—Retinal Fundus Glaucoma Challenge (REFUGE) [32], Online Retinal Fundus Image Database for Glaucoma Analysis and Research (ORIGA) [33], and G1020 [34]—were used for object detection, and one of them, REFUGE, was used for segmentation.

**Figure 4:**
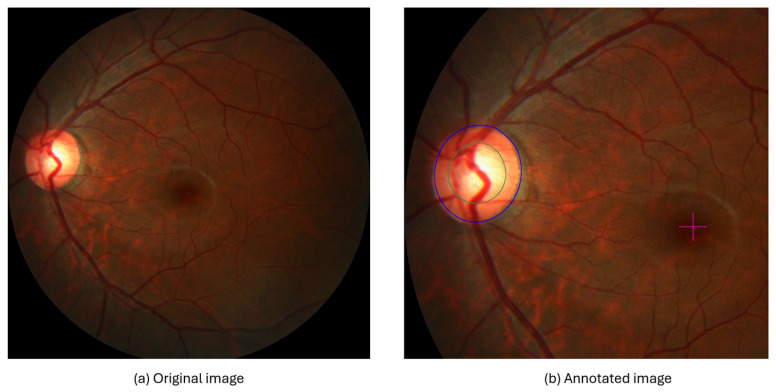
Original fundus image on the left (**a**) and annotated image on the right (**b**), the green represents the OC boundary, blue represents the OD boundary, and the ‘+’ sign indicates the fovea localization.

The REFUGE dataset was created in 2018 for the MICCAI conference, which aimed to compare and evaluate automated techniques presented for glaucoma detection. The dataset consists of 1200 retinal fundus images divided into three sections, each containing 400 images, namely training, testing, and validation. These images were collected from various health centers in China using two devices: a Zeiss Visucam 500 for the training dataset, which had 400 images with a resolution of 2124 × 2056 pixels, and a Canon CR-2 device for the rest of the dataset, which had a resolution of 1634 × 1634 pixels. Then, these images were annotated by seven ophthalmologists and reviewed by one, where four aspects were identified: diagnosis of whether the person had glaucoma, segmentation of both the OD and the OC, and fovea localization. In Figure 4, the original and the annotated images are shown. To diagnose glaucoma, binary labels were used to determine whether the person had glaucoma or was normal. The number of people with glaucoma in the dataset was 120, while the number of normal subjects was 1080. The OD and OC segmentations were displayed as binary segmentations. The fovea location was determined using the coordinates of (x,y) pixel locations [32].

The ORIGA dataset was collected from 2004 to 2007 and became available in 2010. It consists of 650 retinal fundus images collected from the Singapore Malay Eye Study (SiMES), which were annotated by trained professionals from the Singapore Eye Research Institute. It was determined whether each image belonged to a person with glaucoma or without glaucoma. The number of people with glaucoma in the dataset was 168, and 482 did not have glaucoma. Segmentation was performed for the OD and OC, and the CDR value was calculated [33].

The G1020 dataset was collected from 2005 to 2017 and became available in 2020. It consists of 1020 retinal fundus images collected from private clinical practice in Germany, specifically in Kaiserslautern. The dataset represents fundus images taken in routine practice. All images were taken without restrictions, such as the location of the OD. Therefore, the OD location in this dataset covers a wider spatial area than the ORIGA dataset. Annotation was performed using clinical diagnosis, identifying images of individuals with glaucoma and those without. An expert segmented the OD and OC, but the OC was not visible in some images in the dataset. Finally, the results were reviewed by a veteran ophthalmologist with over 25 years of experience. In addition, the vCDR and the four values of the ISNT rule were calculated, along with the bounding box of the OD. The number of people with glaucoma in the dataset was 296, and 724 did not have glaucoma [34]. Table 2 summarizes the datasets used in this research.

### 3.2. Data Pre-Processing

The data were preprocessed differently for each task based on its needs, which will be discussed in detail in the object detection and segmentation sections. However, before starting any task, we prepared the REFUGE dataset. As mentioned earlier in the data collection section, the REFUGE dataset contains three sets, each classified as glaucoma or normal for each image in the dataset, except for the test set. Therefore, we used only the training and validation sets. We used data augmentation techniques only on original glaucoma images and their corresponding masks to increase the dataset size and balance the number of glaucoma and normal images, as each set contains 40 glaucoma and 360 normal images. The techniques we used are as follows [35]:

- Horizontal Flip: The image and mask pixels’ locations are flipped from right to left and vice versa. This flip produces a new image representing the eye opposite the original fundus image as if it were an image of both eyes [36].
- Vertical Flip: The image and mask pixels’ locations are flipped from top to bottom and vice versa. This reversal produces a new image, increasing the sample size [36].
- Horizontal and Vertical Flip: A combination of horizontal and vertical flipping was applied sequentially to the image and mask, resulting in a 180-degree rotation [36].
- Rotation: The images are rotated to any number between 0 and 360 degrees; for example, 90°, 180°, or randomly, either to the right or to the left. The rotation value must be determined based on the nature of the dataset [36]. In this study, we used a random angle within the range of ±10 degrees because rotating the images and masks significantly changes the OD and OC dimensions, affecting the vCDR calculation.
- Horizontal Flip and Rotation: A combination of horizontal flipping and rotation was applied sequentially to the image and mask, with the rotation angle randomly chosen within the range of ±10 degrees.
- Translation: All pixels in the image are moved by a specified number of pixels in one or both axes (*x*-axis and *y*-axis) to generate a new image. In this study, the image and mask were randomly shifted by a value of 1% of the image dimensions in both axes [36]. This small value was chosen because the OD and OC are located at the edges of some images, and a significant shift may result in the removal of the OD and OC from the image.
- Scale (Zoom In and Out): This is the technique of zooming in or out of the image and mask. In this study, the scale was randomly up or down by a value not exceeding 10%. The reason for choosing this small value is that the OD and OC are present at the edges of some images, and a large scale may lead to the removal of the OD and OC from the image.
- Brightness and Contrast Adjustment: This technique is used only on images and is not applied to the mask. It causes changes in pixel values through several techniques. This study applied random changes in image brightness (±20%) and contrast (±10%) to the images [37].

**Figure 5:**
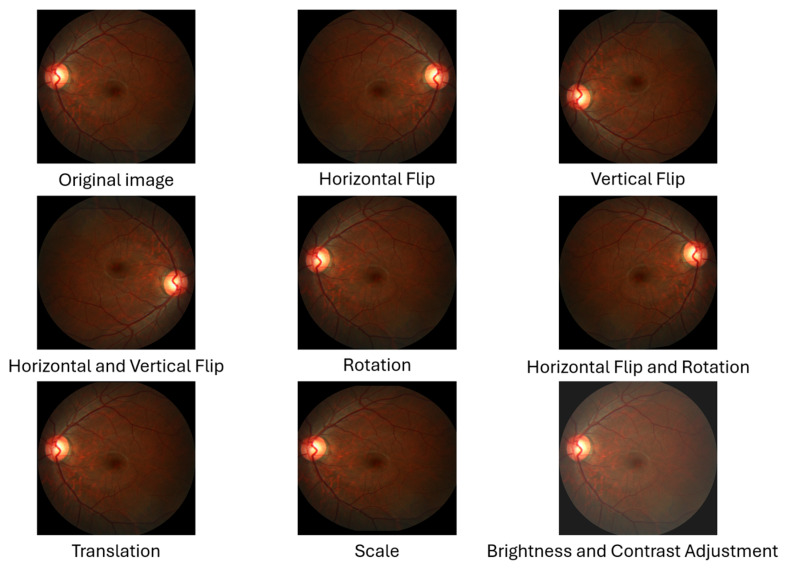
An example of a fundus image from the REFUGE dataset shows the original images without any changes, followed by the remaining images after using eight augmentation techniques to illustrate what the new images added to the dataset look like.

In the augmentation process, we used Albumentations, an open-source Python library known for its speed, flexibility, and ease of use, which offers a broader and more advanced set of transformations than most deep learning frameworks provide [38]. The probability value was set to 1.0 to ensure that augmentations were always applied. As a result, a balance was achieved between the number of glaucoma patients and non-glaucoma patients in the REFUGE dataset, resulting in 720 glaucoma and 720 non-glaucoma images, with a total dataset of 1440 images. Figure 5 shows an example of one of the images after applying augmentation techniques.

### 3.3. Object Detection

Object detection aims to locate the OD from full fundus images and crop them to create a new dataset containing only the region of interest (ROI). This reduces resource consumption and allows focusing work on the important parts without distractions. We used two object detection models: the Faster RCNN model with the ResNet50 backbone, a two-stage detector, and the YOLOv11 one-stage detector to detect the OD from fundus images [39]. We then compared their performance to select the most suitable model for detecting the OD and created a dataset containing cropped OD images. The key steps in the object detection workflow are outlined below.

#### 3.3.1. Dataset Preparation

In this step, three datasets were used for the object detection task: REFUGE after augmentation, ORIGA, and G1020, with a total of 3110 images. We first resized all images to 640 × 640 pixels, as this is one of the preprocessing steps for the YOLOv11 model, which helps the model perform better. The dataset was then divided into three sets: 50% for the training set, 25% for validation, and 25% for testing. The images in the dataset were distributed to reflect the underlying distribution of each classification. The training set, which comprises 50% of the dataset, contains 50% of the total images of glaucoma patients and 50% of the total images of non-glaucoma patients. This ensures a diverse dataset on which the model will be trained and tested.

We used the Computer Vision Annotation Tool (CVAT) [40], an open-source tool to annotate the OD with bounding boxes [41]. Masks were used to illustrate the location and boundaries of the OD in the image during the annotation process to achieve the best results. After completing the annotation, the annotations were exported in two formats: the first for the Faster R-CNN model, which uses the COCO 1.0 format. It is a single JSON file for the entire dataset designed to contain a variety of information, including bounding box information with their absolute pixel values [42]. The second is for the YOLOv11 model, which uses the YOLO 1.1 format. It is a txt file with annotations for each image, containing the class id and bounding boxes that are normalized between 0 and 1 for that image [43].

#### 3.3.2. Training and Evaluation

Both models were trained on the same dataset, with different annotation formats and 100 epochs, which provided a fair comparison. To evaluate object detection models, we used mean average precision (mAP), one of the most popular metrics for evaluating the accuracy of the object detection model. It calculates the average precision (AP), the area under the precision–recall curve for each class, such as the OD class. All AP classes are then used to calculate the average, which is the mAP. There are several Intersection over Union (IoU) thresholds for calculating mAP [44]. For example, mAP 0.5 means a detection is correct if IoU > 0.5 with the ground truth. There is mAP 0.75, meaning a detection is correct if IoU > 0.75. There is also mAP 0.5–0.95, which calculates mAP with multiple IoU thresholds from 0.5 to 0.95. The mean average precision (mAP) can be seen in Equation (1). Table 3 shows the results of precision, recall, mAP 0.5, mAP 0.75, mAP 0.5–0.95, F1-score, and accuracy for the object detection models we used.

(1) $$mAP=\frac{1}{N}\sum_{i=1}^{N}{AP}_{i}$$

Based on the results, we found that Faster R-CNN outperforms YOLOv11 on recall and mAP 0.5, achieving 100%. This means it finds all objects, even detecting the wrong object, and locates them well at 0.5 IoU threshold. In contrast, YOLOv11 leads the rest of the results, achieving 99.87% precision, indicating that the objects found are likely to be the desired ones, meaning that it has high confidence. At mAP 0.75 and mAP 0.5–0.95, it achieved 99.44% and 87.93% better results overall across various IoU thresholds. Regarding the balance between precision and recall, YOLOv11 achieved the best results with an F1-score of 99.87% and an accuracy of 99.74%.

Based on the results, we see that both models achieve good results overall, but the YOLOv11 model outperforms most metrics. Based on the results, we find that YOLOv11′s superiority at mAP 0.75 and mAP 0.5–0.95 indicates high precision, as evidenced by the precision result, as well as a high degree of localization. This is what we need for OD detection. Furthermore, YOLO models are known for their speed due to their structure, which uses a single-stage detector, enabling them to produce results in real time and achieve speed and accuracy without computational complexity. This makes it suitable for use on mobile devices. Our research emphasizes correctly identifying and avoiding false objects as much as possible with good speed. Therefore, YOLOv11 will be used for the OD detection task.

#### 3.3.3. Cropped Region of Interest (ROI)

**Figure 6:**
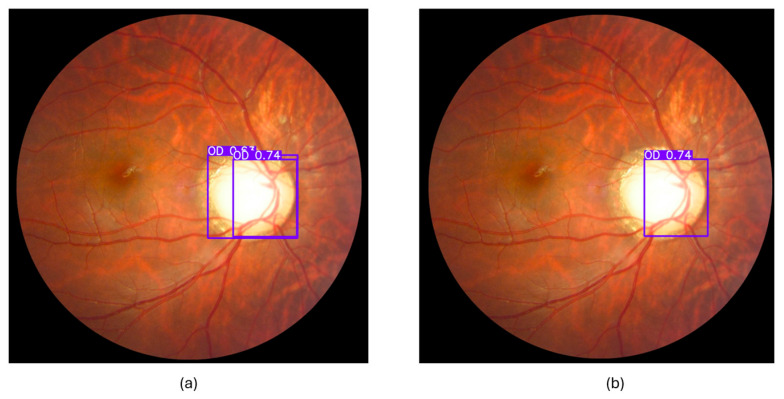
An example of a fundus image using the YOLOv11 model to detect the OD. Image (**a**) shows two bounding boxes when using the default conf = 0.25 and IoU = 0.7. In the image (**b**), the overlap has been removed, leaving the bounding box with the highest conf value after.

Using the YOLOv11 model, we cropped the ROI from the REFUGE dataset and created a dataset containing the cropped ROI. Each image has one OD, so each image should have one bounding box. However, some images had more than one bounding box for the OD. An example of this can be seen in Figure 6. To solve this problem when making predictions using the model, we need to change the value of the Confidence Threshold and IoU Threshold for NMS—Non-Maximum Suppression—as a post-processing step [45]. The Confidence Threshold determines how confident the model is about the presence of an object. To prevent inaccurate predictions, we needed to increase the value of the confidence threshold, so we changed it to conf = 0.5. If the value is high, the model will avoid more objects and is more likely to select the correct object; therefore, 0.5 is the best choice. NMS allows one bounding box for a single object and removes redundancy. This is achieved by selecting the detection with the highest score and removing the remaining detections that overlap with it or are close to it, as these may represent the same object [45]. Therefore, when two bounding boxes overlap with a specified ratio, with NMS IoU = 0.7, it is more likely to have two bounding boxes. However, this number is large, meaning that unless there is an overlap of approximately three-quarters of the object, it will not be processed using NMS, which removes all other bounding boxes and leaves only the one with the highest confidence. Therefore, the value of IoU will be 0.3, which is a small value that allows the removal of other bounding boxes even if the percentage of overlap is small. The result can be seen in Figure 6 after applying the aforementioned changes. There were two bounding boxes in the left image, and now one remains in the right image, which is the one with the highest confidence level.

**Figure 7:**
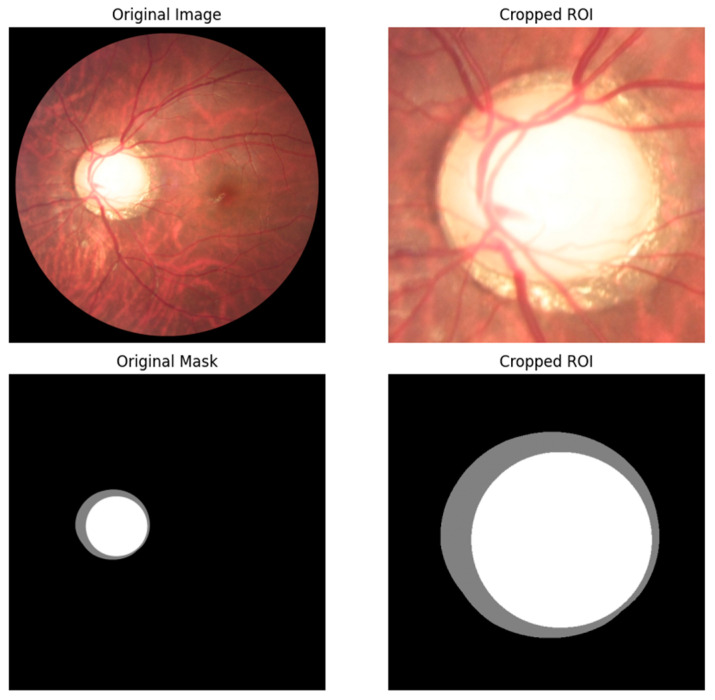
An example of an original fundus image and original mask on the left, and a cropped image and mask on the right after using the YOLOv11 model to detect the OD, adding 70-pixel margins, and standardizing the height and width to the larger value.

After defining one bounding box for each image, the cropping process begins by adding 70-pixel margins in all directions of the predicted bounding box to ensure the full OD is present. The height and width of the bounding box are then measured to determine which is larger and set as the height and width values. This is to ensure that the images are square, not rectangular. This is also because when the images are used in the segmentation process, they will be resized. If the image is not square, the resizing will affect the OD and OC dimensions of the images, and the vCDR value will change. After verifying all measurements for the cropped image, the exact measurements are used to crop the mask, and both are saved as a new dataset. Figure 7 shows the original and cropped images, and the same applies to the mask.

### 3.4. Segmentation and Classification

Detecting glaucoma involves two primary tasks: segmenting the OD and OC to calculate the vCDR and classifying whether an individual has glaucoma based on the value of vCDR. In this step, the cropped REFUGE dataset was used, which was divided into 60% training, 20% evaluation, and 20% testing. We used the Intersection over Union (IoU) and Dice coefficient (DSC) for segmentation evaluation, which measure the overlap between predicted and ground truth segmentation masks. True positives (TP) in the equation represent the common correct pixels between predicted and ground truth masks. Non-common pixels between predicted and ground truth masks are represented by false positives (FP) and false negatives (FN) [46]. The IoU measures the overlap between the prediction and the ground truth. It measures overall performance. To illustrate, we see in Equation (2) that it treats (TP, FN, and FP) equally. This means that a correct prediction contributes equally to the result as an incorrect prediction. This makes it stricter, as it penalizes errors without treating correct predictions differently, as with DSC [47]. DSC is another method for measuring the overlap between the ground truth and the predicted region. To illustrate, we see in Equation (3) that it gives double the weight to correct predictions (TP). In this case, we can see that the effect of TP overlap is larger and more sensitive, while missed pixels (FN) and extra pixels (FP) are treated equally. When an error occurs, it is not penalized as severely, making it more forgiving than IoU. Therefore, to obtain a better picture of performance, more than one metric is used together. For IoU and DSC, the results range between 0, meaning no match, and 1, indicating a perfect match [47]. The accuracy, F1-score, precision, and recall measure classification performance [46]. The models used in the segmentation were CNN-based and transformer-based.

(2) $$IoU=\frac{TP}{TP+FP+FN}$$

(3) $$DSC=\frac{2TP}{2TP+FP+FN}$$

#### 3.4.1. CNN-Based Techniques

We used the U-Net architecture for segmentation because of its known effectiveness in medical image segmentation tasks [48]. Three pre-trained models—ResNet50, VGG16, and MobileNetV2—were used as backbones for U-Net. These models were selected based on their effectiveness in previous studies.

**Figure 8:**
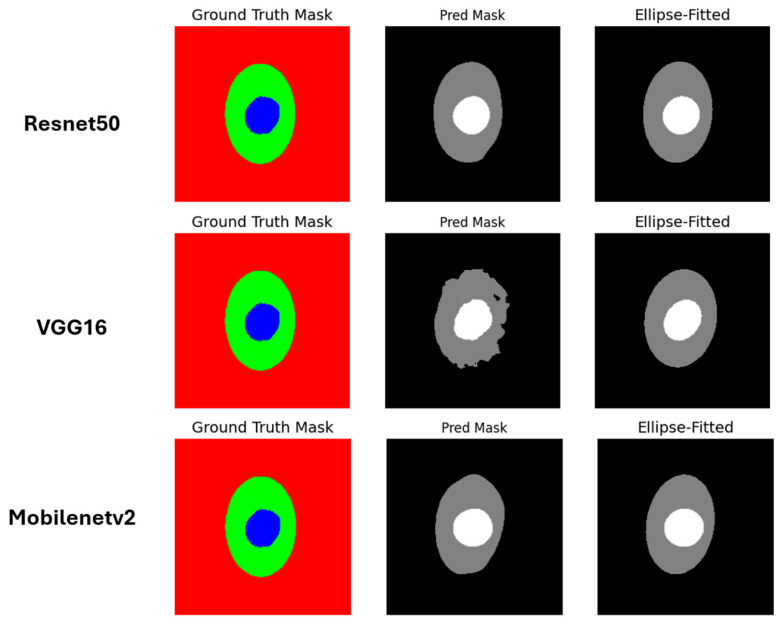
The first image from the left represents the ground truth mask, blue indicates the OC, green indicates the OD, and red indicates the background. The image in the middle represents the result of the predicted mask for each model, and the last image on the right represents the predicted mask for each model after applying ellipse fitting. White indicates the OC, gray indicates the OD, and black indicates the background.

Our implementation is partially based on a publicly available Kaggle notebook [49]. Before segmentation, the images and masks were resized each time based on the pretrained models used to match the size of the model they were trained on. For example, for ResNet50, the image and mask were resized to 224 × 224, and so on for each model. We then prepared the ResNet50 model by using the weights of ImageNet, excluding the classification head, to match our outputs: three classes—background, OD, and OC. Therefore, we used softmax activation. To prepare the training, we used the Adam optimizer and DiceLoss as a loss, training the model for 100 epochs with a batch size of 2. The same steps were followed for VGG16 and MobileNetV2. After training the model, we performed a post-processing technique for segmentation masks, using ellipse fitting for the OD and OC masks. This works by finding the best shape for the ellipses based on a set of points, which improves irregularities and some of the noise, making the shape smoother. In Figure 8, the predicted masks of the three models used before and after ellipse fitting can be seen.

After finding the masks, we calculated the vCDR, IoU, and DSC for each class, along with the mean vCDR error. The classification was based on the vCDR result; if the value was greater than 0.5, the person was classified as having glaucoma. The classification was evaluated using accuracy. All results for U-Net with ResNet50, VGG16, and MobileNetV2 are shown in Table 4. The implementation was carried out in Google Colab using an NVIDIA Tesla T4 GPU (15 GB of memory), CUDA 12.4, system RAM of 12.7 GB, and driver version 550.54.15. Python version ~3.10 and TensorFlow 2.18.0 were used, along with additional libraries, including Segmentation Models (v1.0.1) and Keras Applications (v1.0.8). The U-Net has a different number of parameters based on which backbone is used: ResNet50 contains 20.7 M parameters, MobileNetV2 contains 10.1 M parameters, and VGG16 contains 25.9 M parameters.

#### 3.4.2. Vision Transformer-Based Techniques

We used a MaskFormer model [21] trained on the ADE20k semantic segmentation dataset with Swin-Base as the backbone, obtained from the Hugging Face library [50]. Semantic segmentation was chosen because each fundus image contained one OD and one OC, meaning that only one object existed for each class. The mask uses a per-pixel approach to classify each object. Before using MaskFormer, the dataset must be prepared to suit the model. All images and masks were converted to PIL image type to be in the correct format for MaskFormer. We based our implementation on open-source codebases provided by Hugging Face [51], with some modifications made to suit the structure and purpose of our custom dataset. Glaucoma labels were added to the images and masks for classification and evaluation. We also ensured that the masks were converted to grayscale. This modification required minor changes to the MaskFormer pipeline.

**Figure 9:**
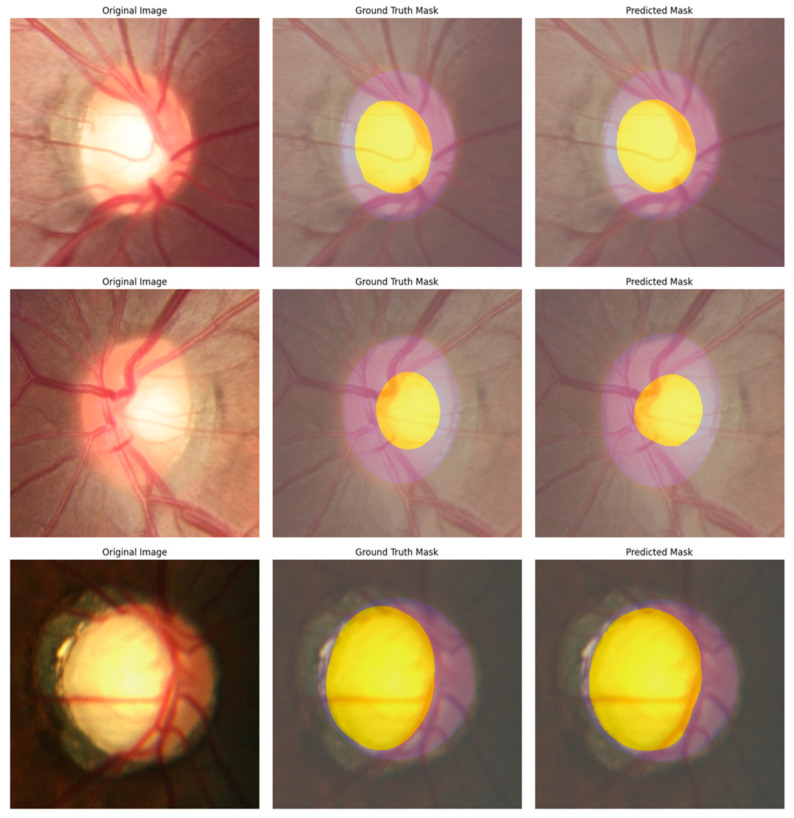
The first image from the left represents the cropped original image, the image in the middle represents the cropped ground truth mask, and the last image on the right is the result of the predicted mask for the model.

Then, we used a preprocessor trained on the ADE20k semantic segmentation dataset with Swin-Base as the backbone to prepare the images as required by the model. In the model definition step, we changed the ID-to-label value to 0 for the background, 1 for the OD, and 2 for the OC. The model was trained on a dataset with 150 classes, while our dataset had three classes. We then changed the ignore mismatched sizes value to true, which replaced the classification head that did not match our work. We used the Adam optimizer and a $5\times 10^{-5}$ learning rate to prepare for training. We trained the model for 100 epochs with a batch size of 2. After training the model, we performed post-processing that was trained on the ADE20k semantic segmentation dataset with Swin-Base as the backbone. We based our implementation on the semantic segmentation tutorial provided by Hugging Face [52], with modifications. In the MaskFormerImageProcessor, we removed ignore_index = 0 to avoid ignoring the background. This also applies to the evaluation metrics for IoU, where ignore_index = 0 was changed to −1 to avoid ignoring the background. Figure 9 presents the segmentation results for three image samples. For each sample, the figure includes the original image, the corresponding ground truth mask, and the predicted mask.

After finding the masks, we calculated the vCDR, mean IoU, and DSC for each class, along with the mean vCDR error. Classification was based on the vCDR result; if the value was greater than 0.5, the person was classified as having glaucoma. The classification was evaluated using accuracy. Table 5 shows the results of MaskFormer trained on the ADE20k semantic segmentation dataset with Swin-Base as the backbone. The implementation was carried out in Google Colab using an L4 GPU, system RAM of 53.0 GB, and Python version ~3, where the MaskFormer model with a Swin backbone contained approximately 101.8 M trainable parameters.

## 4. Results

The results in Table 6 show the CNN-based and vision transformer-based techniques used in this work for the segmentation stages of OD and OC, as well as for classification. The segmentation results were measured using IoU OD, IoU OC, DSC OD, DSC OC, and Mean vCDR Error. Classification was performed using accuracy and F1-score. When comparing the various backbones used with U-Net, we found that MobileNetV2 achieved the best results in all areas. IoU OD, IoU OC, DSC OD, DSC OC, and Mean vCDR Error achieved scores of 84.72%, 85.21%, 91.5%, 91.58%, and 0.03, respectively. In classification based on vCDR values, it achieved an accuracy and F1-score of 87%, followed closely by ResNet50 in all areas.

However, compared to the MaskFormer model using Swin-Base as the backbone with U-Net and MobileNetV2, we found that MaskFormer achieved significantly better results in segmentation. IoU OD, IoU OC, DSC OD, DSC OC, and Mean vCDR Error achieved 88.29%, 91.09%, 93.83%, 93.71%, and 0.02. In classification based on vCDR values, it achieved accuracy and F1-scores of 84.03% and 84.56%, respectively, which were lower than those of MobileNetV2, which achieved 87%. In this study, a comparison was made as fairly as possible between ViT and U-Net architectures using the same dataset and the same split of training, evaluation, and testing sets. The models were trained on the same number of epochs and then the performance was evaluated using the same metrics. Therefore, we saw that, in general, MaskFormer achieved better results in segmentation, with a lower mean vCDR error of 0.02.

Table 7 shows the results of previous studies with the models used in this study for the segmentation and classification stages. Based on the results, we found that in segmentation, MaskFormer outperformed all models in all aspects: IoU OD, IoU OC, DSC OD, and DSC OC, with scores of 88.29%, 91.09%, 93.83%, and 93.71%. However, for the CNN-based models used, the results were inconsistent with previous studies, as MobileNetV2 had the best results after MaskFormer in IoU OC and DSC OC, with scores of 85.21% and 91.58%. However, for IoU OD, ref. [29] achieved better results, with scores of 84.89%, a slight 0.17% increase over MobileNetV2. Additionally, for DSC OD, they achieved better results, with scores of 91.73%, a 0.23% increase over MobileNetV2.

## 5. Discussion

In our research, we proposed a pipeline that starts with the YOLO model to detect ROI regions, which results in a cropped image of the OD region for use in the next steps of OD and OC segmentation, instead of relying on the full fundus image. This is because the focus of the research is on the use of vCDR to detect glaucoma, which is located in the OD region. Using this region reduces the amount of background in the image and allows for more focus on what needs to be segmented. Some studies have found that using a cropped OD image produces better results than using full fundus images [53]. Furthermore, the top three teams participating in the REFUGE Challenge used the cropped versions for OD/OC segmentation [32]. Therefore, the YOLO model was used. This may introduce computational complexity, but it is helpful for model performance. In the real world, full fundus images are often used as input. Therefore, using the YOLO model will help find ROI regions and use a cropped image of the OD region for OD and OC segmentation.

For the evaluation of the performance of several models in OD and OC segmentation and glaucoma classification based on the vCDR, we used U-Net with ResNet50, VGG16, and MobileNetV2 backbones, as well as MaskFormer with a Swin-Base backbone, and compared the performance of these models. As previously explained, MaskFormer achieved the best results in OD and OC segmentation compared to the other models. The performance of these models was then compared with previously published methods.

In a previous study [26], more than one model was used, and the best results were achieved using the Inception V3 model as the backbone of the U-Net architecture for the segmentation of OD and OC. When comparing its results in the segmentation stage with other works, as shown in Table 7, we found that it achieved the lowest results in most aspects among the models, except for DSC OC. However, in the classification stage, which also depends on the same vCDR value we used, a value greater than 0.5 is considered glaucoma. Relying on vCDR for glaucoma diagnosis provides transparency regarding how the model arrived at that diagnosis. They achieved an AUC of 93%, a slight difference of 0.54% more than MaskFormer’s 92.46%. Meanwhile, the F1-score of 74% is very low compared to MaskFormer’s value of 84.56%. Overall, MaskFormer achieved better results using the same classification approach. As for [29], DeepLab v3+ with MobileNet was used for segmentation, achieving results very close to those of U-Net with MobileNetV2, which we used in this work. For classification, they used an ensemble method of pretrained CNNs via transfer learning and SVM, which achieved the highest accuracy and AUC values of 95.59% and 95.10%, respectively, among all models. As for [28], the study also used the ensemble method in both the segmentation and classification stages. However, it did not achieve superiority in either task compared to the other models.

The segmentation results for MaskFormer demonstrate significant advantages in segmentation accuracy, achieving the highest IoU and DSC values across all evaluated methods. While this study focused on glaucoma detection using vCDR as a key biomarker for glaucoma detection, we recognize the diagnostic potential of additional features within fundus images. Thus, in the future, we aim to explore the diagnostic relevance of fine-grained features, such as the pattern and number of small blood vessels entering the OC, to develop a more comprehensive and explainable image-level analysis that integrates these vascular cues rather than relying solely on vCDR.

## 6. Conclusions

This study presents an explainable end-to-end pipeline for automated glaucoma diagnosis from fundus images to assist ophthalmologists in screening processes. The pipeline consists of four stages, starting with object detection of the ROI for the OD using the YOLOv11 model, followed by segmentation of the OD and OC, and the calculation of the vCDR for glaucoma classification. Among the U-Net-based models tested, MobileNetV2 emerged as the most effective backbone. However, the proposed MaskFormer model significantly outperformed all U-Net variants across all segmentation metrics, achieving IoU scores of 88.29% (OD) and 91.09% (OC), and DSC scores of 93.83% (OD) and 93.71% (OC). For the classification task, MaskFormer achieved an accuracy of 84.03% and an F1-score of 84.56%, indicating strong overall performance.

Beyond performance, our proposed framework prioritizes transparency and clinical interpretability, aligning with the growing need for explainable AI in medical diagnostics. By utilizing a well-established structural biomarker of the vCDR, MaskFormer produces clinically meaningful segmentations. This architecture thus offers not only competitive performance but also greater trustworthiness in clinical decision-making. Future work will focus on enhancing classification performance through the integration of full-image contextual features and ensemble strategies while maintaining explainability. Additionally, expanding dataset diversity will further support model generalization and facilitate real-world deployment in ophthalmic screening workflows.

## Figures and Tables

**Table 1:** Summary of related work.

Paper	Dataset	Model	Result	Limitation
[23]	SiMES and SCES	Superpixel classification and support vector machine (SVM)	AUC: SiMES = 80%, SCES = 82.2% AOE *: OD = 9.5%, OC = 24.1%	Lacks generalization across different datasets and is sensitive to image artifacts.
[24]	DRISHTI GS	CNNs with U-Net	Dice score: Disc = 95.05%, Cup = 93.61% IoU: Disc = 90.62%, Cup = 88.09% F1: Disc = 93.56%, Cup 91.62%	Only one dataset was used.
[25]	ORIGA and SCES	Deep learning architecture (M-Net with U-shaped CNN) and polar transformation	AOE: OD = 0.07, OC = 0.23 AUC: ORIGA= 0.85, SCES = 0.90	-
[26]	REFUGE, RIM-ONE r3, and DRISHTI GS.	Inception V3 model as the backbone of the U-Net architecture.	Dice score: 80% IoU: 70% F1: 70%	The result was not better than the previous work and used only two models.
[27]	RIM-One V3 and DRISHTI.	Ensemble network (generalized U-Net and pretrained transfer learning MobileNetV2).	Dice score: Cup 84% to 89% and Disc 92% to 93%, ACC = 88%	Requires more datasets to train ensembles.
[28]	REFUGE and DRISHTI-GS1	Ensemble approach in segmentation using two CNN models (DenseNet201 and ResNet18) and Contrast Limited Adaptive Histogram Equalization (CLAHE)-enhanced inputs	Dice Score: OC = 0.64 OD = 0.88 MAE-CDR = 0.09, Sensitivity = 0.75, Specificity = 0.85 and ROC = 0.856	A small portion of the dataset was used for training, validation, and testing.
[29]	RIM-ONE, ORIGA, DRISHTI GS1, ACRIMA, and REFUGE.	DeepLab v3+ with MobileNet and ensemble method (pre trained CNNs via transfer learning and SVM).	Dice score: 91.73% IoU: 84.89% ACC: 95.59%	Cannot generalize the result, and no segmentation for OC.
[30]	ACRIMA, RIM-ONE, HVD, and Drishti	Classifier Fusion using the maximum voting-based approach (MVB)in Five pre-trained deep CNN models	Two class ACC: 99.57% Three class ACC: 90.55%	Visualization techniques demonstrated have not been measured.
[31]	SMDG-19	Vision Transformer (ViT) and Detection Transformer (DETR)	ACC: DETR = 90.48%, ViT = 87.87%. AUC: DETR = 88%, ViT = 86%.	-

* AOE stands for Average Overlapping Error, Acc stands for Accuracy.

**Table 2:** Dataset summary.

Dataset	Country	Size	Glaucoma	Normal	OD Seg	OC Seg	Cls *
REFUGE [32]	China	1200	120	1080	Yes	Yes	Yes
ORIGA [33]	Singapore	650	168	482	Yes	Yes	Yes
G1020 [34]	Germany	1020	296	724	Yes	Missing OC	Yes

* Cls stands for Classifications.

**Table 3:** Performance results of the object detection models used for OD detection.

Model	Prec	Rec	mAP0.5	mAP0.75	mAP0.5–0.95	F1	Acc
YOLOv11	**99.87%**	99.87%	99.5%	**99.44%**	**87.93%**	**99.87%**	**99.74%**
Faster R-CNN	99.11%	**100%**	**100%**	98%	84.8%	99.55%	99.11%

Numbers in bold represent the best results. Prec stands for Precision, Rec stands for Recall, and Acc stands for Accuracy.

**Table 4:** The results of U-Net segmentation and classification.

U-Net Backbone	Segmentation					Cls *
	IoU OD	IoU OC	DSC OD	DSC OC	Mean vCDR Error	Accuracy
ResNet50	84.22%	85.02%	91.14%	91.47%	**0.03**	86%
VGG16	77.31%	79.92%	86.57%	88.46%	0.05	84%
Mobilenetv2	**84.72%**	**85.21%**	**91.5%**	**91.58%**	**0.03**	**87%**

* Cls stands for classification. Numbers in bold represent the best results.

**Table 5:** The results of MaskFormer segmentation and classification.

MaskFormer Backbone	Segmentation				Classification			
	Mean IoU	IoU OD	IoU OC	Mean vCDR Error	Acc	Prec	Rec	F1
Swin-Base	92.51%	88.29%	91.09%	0.02	84.03%	80.77%	88.73%	84.56%

Prec stands for Precision, Rec stands for Recall, and Acc stands for Accuracy.

**Table 6:** The results of U-Net and MaskFormer segmentation and classification.

Model	Segmentation					Classification	
	IoU OD	IoU OC	DSC OD	DSC OC	Mean vCDR	Acc	F1
U-net + ResNet50	84.22%	85.02%	91.14%	91.47%	0.03	86%	86%
U-net + VGG16	77.31%	79.92%	86.57%	88.46%	0.05	84%	85%
U-net + MobileNetV2	84.72%	85.21%	91.5%	91.58%	0.03	**87%**	**87%**
MaskFormer + Swin-Base	**88.29%**	**91.09%**	**93.83%**	**93.71%**	**0.02**	84.03%	84.56%

Numbers in bold represent the best results.

**Table 7:** Comparison of U-Net and MaskFormer results in this research with previous studies on glaucoma segmentation and classification.

Model	Dataset	Segmentation				Classification	
		IoU OD	IoU OC	DSC OD	DSC OC	Acc	AUC
[28]	REFUGE, DRISHTI-GS1	-	-	88%	64%	-	85%
[29]	REFUGE, RIM-ONE, DRISHTI-GS1, ORGIA, ACRIMA	84.89%	-	91.73%	-	**95.59%**	**95.10%**
[26]	REFUGE, RIM-ONE r3, DRISHTI-GS	73% (±18)	72% (±17)	83% (±17)	82% (±15)	**-**	93%
U-net + ResNet50	REFUGE	84.22%	85.02%	91.14%	91.47%	86%	-
U-net + VGG16	REFUGE	77.31%	79.92%	86.57%	88.46%	84%	-
U-net + MobileNetV2	REFUGE	84.72%	85.21%	91.5%	91.58%	87%	-
MaskFormer + Swin-Base	REFUGE	**88.29%**	**91.09%**	**93.83%**	**93.71%**	84.03%	92.46%

Numbers in bold represent the best results.

## Data Availability

In this study, publicly available datasets from Kaggle were used. These data can be found here: https://www.kaggle.com/datasets/arnavjain1/glaucoma-datasets, accessed on 12 March 2024.

## Abbreviations

The following abbreviations are used in this manuscript:

ONH	Optic Nerve Head
OD	Optic Disc
OC	Optic Cup
IOP	Intraocular Pressure
WHO	World Health Organization
CDR	Cup-to-Disc Ratio
vCDR	Vertical Cup-to-Disc Ratio
VF	Visual Fields
OCT	Optical Coherence Tomography
CAD	Computer-Aided Diagnosis
NLP	Natural Language Processing
ViTs	Vision Transformers
DETR	Detection Transformer
CNNs	Convolutional Neural Networks
ML	Machine Learning
DL	Deep Learning
AI	Artificial Intelligence
XAI	Explainable Artificial Intelligence
TL	Transfer Learning
Grad-CAM	Gradient-Weighted Class Activation Mapping
LIME	Locally Interpretable Model-Agnostic Explanations
REFUGE	Retinal Fundus Glaucoma Challenge
ORIGA	Online Retinal Fundus Image Database for Glaucoma Analysis and Research
ROI	Region of Interest
SiMES	Singapore Malay Eye Study
CVAT	Computer Vision Annotation Tool
YOLO	You Only Look Once
RPN	Region Proposal Network
SVM	Support Vector Machine
CLAHE	Contrast Limited Adaptive Histogram Equalization
mAP	Mean Average Precision
AP	Average Precision
NMS	Non-Maximum Suppression
IoU	Intersection over Union
DSC	Dice coefficient
MVB	Maximum Voting-Based
TP	True Positives
FP	False Positives
FN	False Negatives
W-MSA	Window-Based Multi-Head Self-Attention
SW-MSA	Shifted Window-Based Multi-Head Self-Attention
